# Sociodemographic and physical predictors of non-participation in community based physical checkup among older neighbors: a case-control study from the Kyoto-Kameoka longitudinal study, Japan

**DOI:** 10.1186/s12889-018-5426-5

**Published:** 2018-05-02

**Authors:** Miwa Yamaguchi, Tsukasa Yoshida, Yosuke Yamada, Yuya Watanabe, Hinako Nanri, Keiichi Yokoyama, Heiwa Date, Motoko Miyake, Aya Itoi, Emi Yamagata, Taeko Masumoto, Yasuko Okayama, Yasuko Yoshinaka, Misaka Kimura, Kyoto-Kameoka study group

**Affiliations:** 1 Department of Nutrition and Metabolism, National Institutes of Biomedical Innovation, Health and Nutrition, Tokyo, 162-8636 Japan; 2 Senior Citizen’s Welfare Section, Kameoka City Government, Kyoto, 621-8501 Japan; 3 Laboratory of Applied Health Sciences, Kyoto Prefectural University of Medicine, Kyoto, 602-8566 Japan; 4 Faculty of Economics and Business Administration, Kyoto Gakuen University, Kyoto, 621-8555 Japan; 5 Faculty of Health and Sports Science, Doshisha Unviersity, Kyoto, 610-0394 Japan; 6 Department of Health and Sports Sciences, Kyoto Gakuen University, Kyoto, 621-8555 Japan; 7 Faculty of Data Science, Shiga University, Shiga, 522-8522 Japan; 8 Department of Health, Sports and Nutrition, Faculty of Health and Welfare, Kobe Women’s University, Hyogo, 650-0046 Japan; 9 Faculty of Nursing, Doshisha Women’s College of Liberal Arts, Kyoto, 610-0395 Japan

**Keywords:** Physical activity program, Sociodemographic factors, Physical frailty, Participation rate, Community-dwelling older people, Health promotion, Case-control study

## Abstract

**Background:**

It is difficult to obtain detailed information on non-participants in physical and health examination checkups in community-based epidemiological studies. We investigated the characteristics of non-participants in a physical and health examination checkup for older adults in a nested study from the Japanese Kyoto-Kameoka Longitudinal Study.

**Methods:**

We approached a total of 4831 people aged ≥65 years in 10 randomly selected intervention regions. Participants responded to a mail-based population survey on needs in the sphere of daily life to encourage participation in a free face-to-face physical checkup examination; 1463 participants (706 men, 757 women) participated in the physical checkup. A multiple logistic regression model was performed to investigate the adjusted odds ratios (aOR) of non-participation based on sociodemographic status apart from psychological and physiological frailty as assessed by the validated Kihon Checklist.

**Results:**

There was a significant, inverse relationship between non-participation and frequently spending time alone among individuals who lived with someone or other family structure (aOR = 0.53, standard error [SE] 0.08 in men, aOR = 0.66, SE 0.09 in women). Very elderly (over 80 years old) women, poorer health consciousness and current smoking in both sexes and poor self-rated health in men, were significantly related to higher non-participation rates. In both sexes, individuals who did not participate in community activities were significantly more likely to be non-participants than individuals who did (aOR = 1.94, SE 0.23 in men, aOR = 3.29, SE 0.39 in women). Having low IADL and physical functioning scores were also associated with higher rates of non-participation.

**Conclusion:**

Health consciousness and lack of community activity participation were predictors of non-participation in a physical checkup examination among older adults. In addition, lower IADL and physical functioning/strength were also predictors of non-participation.

On the contrary, older inhabitants living with someone tended to participate in the physical checkup examination for social interchange when they were frequently alone in the household. This study suggests the importance of considering aging especially for women and poor sociodemographic background and physical frailty for both sexes so that older people can access health programs without difficulty.

**Trial registration:**

UMIN000008105. Registered 26 April 2012. Retrospectively registered.

**Electronic supplementary material:**

The online version of this article (10.1186/s12889-018-5426-5) contains supplementary material, which is available to authorized users.

## Background

In 2014, the life expectancy at birth was on average 86.8 years for women and 80.5 years for men in Japan [1]. In line with the fact that Japanese life expectancy is the highest in the world, the total amount of nursing care costs in fiscal year 2013 was 9.2 trillion yen (81 billion USD), due to the aging of Japanese society [1]. The Japanese government encouraged the promotion of community based multi-faceted approaches to prevent long-term care in the Integrated Community Care System [2].

The importance of lifestyle interventions in preventing long-term care is well-known [3–5]. Several studies investigating life-style interventions for independent life for older people have reported positive effects on physical ability [6–8], well-being [7, 9], and mental well-being [9]. A previous article reported that a lifestyle-based physical activity program resulted in a 640.4 USD/year lower cost of total medical expenditure in the intervention group as compared to the control group [10]. However, one review article indicated that the initial participation level in physical activity programs for people aged 55 years or older ranged from merely 1.4 to 16.2% (mean = 9.2%), among five programs with durations ranging 1 to 6 months [11]. Another review article indicated that the very elderly, older people from black and minority ethnic groups, and older people living in deprived areas encountered barriers to being recruited for and engaging in health promotion interventions and related research [12, 13]. One previous Japanese study provided evidence that non-regular participants who attended a sports group once or twice a month, a few times a year, or never were more likely to have certain sociodemographic (i.e., lower educational level, being employed, and having worked primarily in the agricultural/forestry/fishery industry) and biopsychosocial characteristics (i.e., poor self-rated health and depression) [14]. Therefore, it is important to consider social and environmental support so that older people can engage in health promotion interventions [3, 5, 13]. However, previous reviews have indicated that there is still no high-quality evidence regarding the effectiveness of different approaches for older people who experience barriers to enrolling in health programs, including those in Japan [5, 13]. We hypothesize that poor sociodemographic, physical, and psychological factors may discourage older inhabitants from participating in community-based health programs. Thus, we examined the characteristics of non-participants compared with participants in the intervention program, including sociodemographic, physical, and psychological factors in community-based health programs in Japan.

## Methods

### Study participants

**Fig. 1:**
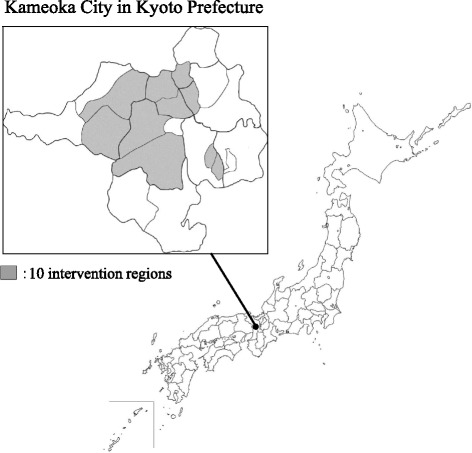
Geographical features of Kameoka city, Kyoto Prefecture, Japan

**Fig. 2:**
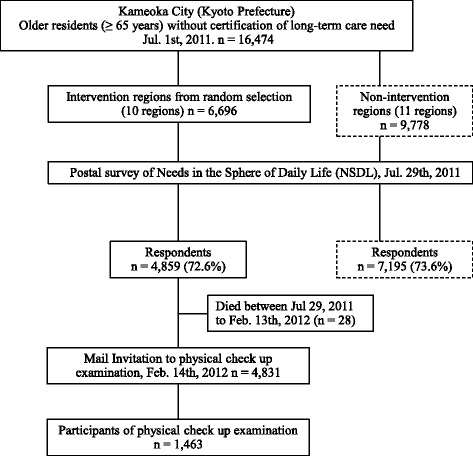
Flow chart of the physical checkup in the Kyoto-Kameoka study

Kameoka city is located in the central area of Kyoto Prefecture, about 25 km west of Kyoto City (Fig. 1) [15]. The Kyoto-Kameoka Longitudinal Study in the city has been described previously [16]. This study has been conducted since July 29^th^, 2011 with the Needs in the Sphere of Daily Life (NSDL) survey created by the national government’s Ministry of Health, Labour and Welfare, modified for the present study [16], as a baseline (Fig. 2). The NSDL survey was given to 16,474 people aged 65 years or older without indication of long-term care need that was registered by the city office. From the 12,054 responders, the research office formally mailed an invitation for a free physical checkup examination in February 14^th^, 2012 to the 4831 responders (28 of 4859 residents died before the physical checkup) who lived in 10 randomly selected intervention regions, from a total of 21 regions. The physical checkup was a one to three-day event in each region at the community center, depending on the region’s population, as part of the population-based comprehensive geriatric intervention program for preventing long-term care. The flyer described the checkup duration (approximately one hour of total) and measurement items to assess their mobility ability and fitness, including: right front thigh muscle thickness, grip strength, maximum isometric right knee extension strength, and their usual and maximum physical functions. After the invitation, 1463 participants (participation rates 31.6% in men, 28.9% in women) voluntarily participated in the physical checkup. Considering the distribution, age-adjusted participation rates were 31.8% in men and 27.4% in women, calculated by multiplying crude participation rates and the population of Kameoka city (taken from the population statistics in July 1^st^, 2011 [17, 18] among each age category (65–69, 70–74, 75–79, and ≥80 years) in men and women. Therefore, the final sample comprised 3368 people (1509 men, 1859 women) in the case group of non-participants in the physical checkup, and 1463 participants (706 men, 757 women) in the control group who did participate.

### Explanatory variables

Sociodemographic variables proposed to be social determinants of health were employed as explanatory variables from the self-reported questionnaire (Additional file 1: Table S1) [18]. First, age, calculated from the resident’s date of birth registered in the city office, was classified into bins of 65–69, 70–74, 75–79, and ≥ 80 years. Social status variables included family structure (with someone or others / living alone), living with spouse (yes / no), alone at home: spending a day at home alone all day (rarely / frequently), and educational attainment (≥13, 10–12, or ≤9 years). The questions regarding “living with spouse” and “alone at home” were only for people who reported living with someone or others. Here in Japan, most citizens can receive the National Pension (basic pension) after they turn 65 years old. According to their occupations, residents are classified into three levels of the National Pension: Category 1 is for residents aged 20 to 59 years who are not Category 2 or 3 insured persons (e.g., self-employed persons, farmers, or students); Category 2 combines Employees’ Pension Insurance for private company workers or Mutual Aid Pension for public employees and private school teachers and employees with the basic pension; and Category 3 is for dependents of the spouse who belongs to insured Category 2 [19]. In this study, we grouped pension levels into three categories: the National Pension (Category 1), the Employees’ Pension (Categories 2 and 3), and no pension or other pensions. Other economic variables examined included (subjective) economic difficulty (yes / no), current employment (working / not working), and automobile access (yes / no). Health-related questions included: interest in health topics (yes / no), self-rated health (good / poor), and smoking status (never, past, and current). To assess whether an individual participates in community activities, we coded this variable as “yes” when residents answered affirmatively to participating in at least one of the following activities: local events (e.g., festivals and dances), community associations, hobby activity groups, senior citizen clubs, volunteer groups, and other groups. Population density (city ≥1000 / province <1,000 people/km^2^) was also assessed.

### Assessment of frailty by the Kihon checklist

The Kihon Checklist (KCL) is a well-validated tool for the screening of long-term care needs for community-dwelling older residents who have difficulties with self-support. In Japan, the KCL has being widely used to assess frailty [20, 21]. The KCL consists of 25 items and has seven sections. A total KCL score of ≥7 points was defined as suggesting frailty and a high risk of needing long-term care (Additional file 2: Table S2). Each of the seven sections of KCL also have cut-off points for assessing frailty based on norms proposed by previous reports [22–24]: ≥3 for instrumental activities of daily living (IADL), ≥3 for physical function/strength, 2 for malnutrition, ≥2 for oral function, ≥1 for socialization (being housebound), ≥1 for memory, and  ≥2 for mood.

### Statistical analysis

To analyze differences in the characteristics of non-participation versus participation in the physical checkup, we used a chi-square test for categorical variables. In consideration of the gender difference in participation rates, we analyzed separately by gender. We also estimated the adjusted odds ratios (aOR) and 95% confidence intervals (CI) for non-participation using a multiple logistic regression model. Possible sociodemographic factors for non-participation of health checkup were extracted: age (65–69, 70–74, 75–79, and  ≥80 years), social status (family structure, living with spouse, alone at home, and educational attainment), economic status (economic difficulty, pension, current employment, and automobile access), health conscious (interest in health topics, self-rated health, and smoking status), community activities (yes / no), and population density (city / province). In Model 1, analyses for each variable were adjusted for all sociodemographic variables we collected. In Model 2, the total score for the KCL was added to Model 1. The association of non-participation with seven types of frailty (IADL, function/strength, malnutrition, oral function, socialization, memory, and mood) was analyzed in addition to the total KCL score. In the analysis with the KCL score, Model 1 was adjusted for all sociodemographic variables. The seven types of KCL were added to Model 1 in Model 2, in order to investigate which types were primarily associated with non-participation. All extracted factors were categorical variables. We used unknown (missing) variables including either merely missing or “no response,” purposefully. The variables estimated the aORs of non-participation in each sociodemographic status and frailty level to compare better sociodemographic status and lower frailty levels as the reference groups. Calculations and statistical tests were performed using the STATA software, version 14.0 (StataCorp LP, College Station, TX). All statistical tests were 2-sided and *p*-values <0.05 were considered statistically significant.

## Results

As can be seen in Table 1, the proportion of non-participation in women was higher than men (non-participation 1509, 68.1% in men; 1859, 71.1% in women). The proportions of non-participation of those in lower socioeconomic groups had a tendency to be higher than that of those in the higher socioeconomic groups (Table 1).

**Table 1:** Sociodemographic characteristics of physical checkup non-participants and participants among Japanese older men and women

	Men (n = 2,215)			Women (n = 2,616)		
	Non-participation	Participation		Non-participation	Participation	
	n (%)	n (%)	*P*-value ^a^	n (%)	n (%)	*P*-value ^a^
Population	1509 (68.1)	706 (31.9)	-	1859 (71.1)	757 (28.9)	-
Age (years)						
65–69	519 (34.4)	226 (32.0)	0.496	543 (29.2)	246 (32.5)	<0.001^**^
70–74	411 (27.2)	197 (27.9)		439 (23.6)	243 (32.1)	
75–79	297 (19.7)	156 (22.1)		387 (20.8)	173 (22.9)	
≥80	282 (18.7)	127 (18.0)		490 (26.4)	95 (12.6)	
Social status						
Family structure						
With someone or others	1292 (85.6)	630 (89.2)	0.026^*^	1454 (78.2)	594 (78.5)	0.021^*^
Alone	85 (5.6)	37 (5.2)		243 (13.1)	118 (15.6)	
Unknown ^d^	132 (8.8)	39 (5.5)		162 (8.7)	45 (5.9)	
Living with spouse ^b^						
Yes	413 (29.0)	199 (29.8)	<0.001^**^	572 (35.4)	234 (36.6)	<0.001^**^
No	773 (54.3)	411 (61.4)		770 (47.7)	342 (53.5)	
Unknown	238 (16.7)	59 (8.8)		274 (17.0)	63 (9.9)	
Alone at home ^b^						
Rarely	294 (20.7)	81 (12.1)	<0.001^**^	1078 (66.7)	477 (74.7)	0.001^*^
Frequently	896 (62.9)	503 (75.2)		276 (17.1)	86 (13.5)	
Unknown	234 (16.4)	85 (12.7)		262 (16.2)	76 (11.9)	
Educational attainment (years)						
≥13	407 (27.0)	137 (19.4)	<0.001^*^	270 (14.5)	131 (17.3)	<0.001^**^
10–12	541 (35.9)	297 (42.1)		793 (42.7)	382 (50.5)	
≤9	334 (22.1)	211 (29.9)		531 (28.6)	161 (21.3)	
Unknown	227 (15.0)	61 (8.6)		265 (14.3)	83 (11.0)	
Economic status						
Economic difficulty						
Yes	1003 (66.5)	428 (60.6)	0.002^*^	1156 (62.2)	442 (58.4)	0.085
No	440 (29.2)	257 (36.4)		585 (31.5)	272 (35.9)	
Unknown	66 (4.4)	21 (3.0)		118 (6.4)	43 (5.7)	
Pension						
National Pension	440 (29.2)	161 (22.8)	<0.001	1163 (62.6)	475 (62.8)	0.010^*^
Employees’ Pension	970 (64.3)	528 (74.8)		537 (28.9)	240 (31.7)	
Nothing or others	57 (3.8)	9 (1.3)		93 (5.0)	17 (2.3)	
Unknown	42 (2.8)	8 (1.1)		66 (3.6)	25 (3.3)	
Current employment						
Working	442 (29.3)	201 (28.5)	0.110	191 (10.3)	100 (13.2)	0.054
Not working	949 (62.9)	466 (66.0)		1485 (79.9)	595 (78.6)	
Unknown	118 (7.8)	39 (5.5)		183 (9.8)	62 (8.2)	
Automobile access						
Yes	1214 (80.5)	615 (87.1)	<0.001^**^	1211 (65.1)	519 (68.6)	0.094
No	295 (19.6)	91 (12.9)		648 (34.9)	239 (31.4)	
Health consciousness						
Interest in health topics						
Yes	1221 (80.9)	636 (90.1)	<0.001^**^	1672 (89.9)	722 (95.4)	<0.001^**^
No	240 (15.9)	59 (8.4)		140 (7.5)	21 (2.8)	
Unknown	48 (3.2)	11 (1.6)		47 (2.5)	14 (1.9)	
Self-rated health						
Good	1097 (72.7)	583 (82.6)	<0.001^**^	1396 (75.1)	633 (83.6)	<0.001^**^
Poor	346 (22.9)	92 (13.0)		387 (20.8)	97 (12.8)	
Unknown	66 (4.4)	31 (4.4)		76 (4.1)	27 (3.6)	
Smoking status						
Never	329 (21.8)	163 (23.1)	0.001^*^	1547 (83.2)	666 (88.0)	0.001^*^
Past	794 (52.6)	415 (58.8)		111 (6.0)	45 (5.9)	
Current	316 (20.9)	99 (14.0)		87 (4.7)	14 (1.9)	
Unknown	70 (4.6)	29 (4.1)		114 (6.1)	32 (4.2)	
Community activities						
Yes	915 (60.6)	543 (76.9)	<0.001^**^	1062 (57.1)	632 (83.5)	<0.001^**^
No	594 (39.4)	163 (23.1)		797 (42.9)	125 (16.5)	
Population density ^c^						
City	534 (35.4)	296 (41.9)	0.003	661 (35.6)	297 (39.2)	0.077
Province	975 (64.6)	410 (58.1)		1198 (64.4)	460 (60.8)	

Non-participation, non-participation of physical checkup; participation, participation of health checkup

^*^p-value <0.05, ^**^p-value <0.001

^a^Chi-square test was performed for categorical variables

^b^These results were indicated among those who were ‘living with someone or others’ in the family structure (non-participation 1,424 in men, 1,616 in women; physical checkup 669 in men, 639 in women)

^c^Population density (people/km^2^) was categorized as city (≥1,000) and province (<1,000)

^d^All unknown (missing) variables were included either merely missing or “no response” on purpose

As shown in Table 2, older adults with higher total and sub-score KCL, defined frailty, were more likely to be non-participants, regardless of sex (Table 2).

**Table 2:** Kihon Checklist scores among physical checkup non-participants and participants among Japanese older men and women

	Men			Women		
	Non-participation	Participation		Non-participation	Participation	
Kihon Checklist	n (%)	n (%)	*P*-value ^a^	n (%)	n (%)	*P*-value ^a^
Total score						
<7	671 (44.5)	390 (55.2)	<0.001^**^	725 (38.9)	413 (54.6)	<0.001^**^
≥7	343 (22.7)	148 (21.0)		470 (25.2)	128 (16.9)	
Unknown ^b^	495 (32.8)	169 (23.8)		671 (36.0)	216 (28.5)	
IADL						
<3	1102 (73.0)	603 (85.4)	<0.001^**^	1406 (75.4)	694 (91.7)	<0.001^**^
≥3	311 (20.6)	79 (11.2)		313 (16.8)	31 (4.1)	
Unknown	96 (6.4)	24 (3.4)		147 (7.9)	32 (4.2)	
Physical function/strength						
<3	1075 (71.2)	576 (81.6)	<0.001^**^	1075 (57.6)	539 (71.2)	<0.001^**^
≥3	262 (17.4)	74 (10.5)		561 (30.1)	139 (18.4)	
Unknown	172 (11.4)	56 (7.9)		230 (12.3)	79 (10.4)	
Malnutrition						
<2	1225 (81.2)	619 (87.7)	<0.001^**^	1467 (78.6)	648 (85.6)	<0.001^**^
2	36 (2.4)	11 (1.6)		41 (2.2)	14 (1.9)	
Unknown	248 (16.4)	76 (10.8)		358 (19.2)	95 (12.6)	
Oral function						
<2	1048 (69.5)	530 (75.1)	0.010^*^	1312 (70.3)	576 (76.1)	0.003^*^
≥2	377 (25.0)	152 (21.5)		438 (23.5)	154 (20.3)	
Unknown	84 (5.6)	24 (3.4)		116 (6.2)	27 (3.6)	
Socialization						
<1	960 (63.6)	479 (67.9)	0.075	996 (53.4)	512 (67.6)	<0.001^**^
≥1	487 (32.3)	208 (29.5)		793 (42.5)	225 (29.7)	
Unknown	62 (4.1)	19 (2.7)		77 (4.1)	20 (2.6)	
Memory						
≥1	780 (51.7)	434 (61.5)	<0.001^**^	672 (36.0)	220 (29.1)	<0.001^**^
<1	642 (42.5)	251 (35.6)		1097 (58.8)	507 (67.0)	
Unknown	87 (5.8)	21 (3.0)		97 (5.2)	30 (4.0)	
Mood						
<2	911 (60.4)	502 (71.0)	<0.001^**^	1008 (54.0)	500 (66.1)	<0.001^**^
≥2	399 (26.4)	148 (21.0)		563 (30.2)	179 (23.7)	
Unknown	199 (13.2)	56 (7.9)		295 (15.8)	78 (10.3)	

*IADL* instrumental activities of daily living

^*^p-value <0.05, ^**^ *p*-value <0.001

^a^Chi-square test was performed for categorical variables

^b^All unknown (missing) variables were included either merely missing or “no response” on purpose

Tables 3 and 4 show the adjusted odds ratios for non-participation per sociodemographic status among men and women, respectively. First, 75–79-year-old men had a significantly lower aOR of non-participation compared to that of the lowest age group (65–69 years) in the fully adjusted Model 2 (Table 3). Among men, we observed significant, inverse associations between ‘frequent’ group in the alone at home and non-participation, as compared to ‘rarely’ group (aOR = 0.53, 95% CI: 0.40, 0.70) (Table 3). Individuals with ≤9 years of educational attainment were more likely to be non-participants (aOR = 1.52, 95% CI: 1.15, 2.01) compared those with ≥13 years. In terms of significant economic predictors, being on ‘the National Pension’ compared to ‘the Employees’ Pension’ and ‘No’ group of automobile access indicated more likely to be a non-participant (aOR = 1.30, 95% CI: 1.04, 1.63 in the National Pension; aOR =1.32, 95% CI: 1.00, 1.74 in automobile access). Having lower levels of all three types of health consciousness showed significantly positive associations with non-participation (aOR = 1.69, 95% CI: 1.23, 2.33 for ‘No’ of interest in health topics; aOR =1.68, 95% CI: 1.27, 2.22 for ‘poor’ in self-rated health; aOR = 1.62, 95% CI: 1.19, 2.21 for ‘current’ in smoking status). Individuals who were not engaged in community activities, ‘No’ group, were more likely to be non-participants, compared to than those who did not engage in any such activities (aOR = 1.94, 95% CI: 1.54, 2.44).

**Table 3:** The adjusted odds ratios for physical check-up non-participation per sociodemographic status variables among older men

	Non-participation	Model 1 ^c^	Model 2 ^d^
	/ total, n (%)	aOR (95% CI)	aOR (95% CI)
Age (years)			
65–69	519/745 (69.7)	reference	reference
70–74	411/608 (67.6)	0.88 (0.69, 1.12)	0.88 (0.69, 1.12)
75–79	297/453 (65.6)	0.75 (0.57, 0.99)^*^	0.73 (0.56, 0.96)^*^
≥80	282/409 (69.0)	0.82 (0.61, 1.09)	0.79 (0.58, 1.07)
Social status			
Family structure			
With someone or others	1292/1922 (67.2)	reference	reference
Alone	85/122 (69.7)	1.00 (0.65, 1.52)	0.98 (0.64, 1.49)
Unknown ^a^	132/171 (77.2)	1.39 (0.93, 2.06)	1.33 (0.89, 1.99)
Living with spouse ^d^			
Yes	413/612 (67.5)	reference	reference
No	773/1184 (65.3)	0.97 (0.78, 1.21)	0.98 (0.79, 1.23)
Unknown	238/297 (80.1)	1.92 (1.12, 3.29)^*^	1.91 (1.11, 3.28)^*^
Alone at home ^d^			
Rarely	294/375 (78.4)	reference	reference
Frequently	896/1399 (64.1)	0.52 (0.39, 0.69)^**^	0.53 (0.40, 0.70)^**^
Unknown	234/319 (73.4)	0.53 (0.33, 0.83)^*^	0.53 (0.33, 0.83)^*^
Educational attainment (years)			
≥13	334/545 (61.3)	reference	reference
10–12	541/838 (64.6)	1.04 (0.83, 1.32)	1.04 (0.82, 1.31)
≤9	407/544 (74.8)	1.53 (1.16, 2.02)^*^	1.52 (1.15, 2.01)^*^
Unknown	227/288 (78.8)	1.85 (1.30, 2.65)^*^	1.74 (1.21, 2.50)^*^
Economic status			
Economic difficulty			
No	440/697 (63.1)	reference	reference
Yes	1003/1431 (70.1)	1.19 (0.96, 1.46)	1.20 (0.98, 1.48)
Unknown	66/87 (75.9)	1.34 (0.76, 2.39)	1.31 (0.73, 2.32)
Pension			
National Pension	970/1498 (64.8)	reference	reference
Employees’ Pension	440/601 (73.2)	1.32 (1.05, 1.65)^*^	1.30 (1.04, 1.63)^*^
Nothing or others	57/66 (86.4)	1.96 (0.93, 4.11)	2.01 (0.96, 4.23)
Unknown	42/50 (84.0)	1.78 (0.76, 4.18)	1.88 (0.80, 4.42)
Current employment			
Working	442/643 (68.7)	reference	reference
Not working	949/1415 (67.1)	0.81 (0.65, 1.02)	0.82 (0.66, 1.02)
Unknown	118/157 (75.2)	0.90 (0.58, 1.41)	0.87 (0.56, 1.36)
Automobile access			
Yes	1214/1829 (66.4)	reference	reference
No	295/386 (76.4)	1.36 (1.03, 1.78)^*^	1.32 (1.00, 1.74)^*^
Health conscious			
Interest in health topics			
Yes	1221/1857 (65.8)	reference	reference
No	240/299 (80.3)	1.64 (1.20, 2.24)^*^	1.69 (1.23, 2.33)^*^
Unknown	48/59 (82.0)	2.00 (1.01, 4.00)^*^	1.69 (0.83, 3.43)
Self-rated health			
Good	1097/1680 (65.3)	reference	reference
Poor	346/438 (79.0)	1.62 (1.24, 2.12)^**^	1.68 (1.27, 2.22)^**^
Unknown	66/97 (68.0)	0.66 (0.33, 1.35)	0.63 (0.31, 1.28)
Smoking status			
Never	329/492 (66.9)	reference	reference
Past	794/1209 (65.7)	0.98 (0.78, 1.24)	0.98 (0.78, 1.24)
Current	316/415 (76.1)	1.63 (1.19, 2.21)^*^	1.62 (1.19, 2.21)^*^
Unknown	70/99 (70.7)	1.04 (0.50, 2.17)	0.92 (0.44, 1.93)
Community activities			
Yes	915/1458 (62.8)	reference	reference
No	594/757 (78.5)	1.94 (1.54, 2.43)^**^	1.94 (1.54, 2.44)^**^
Population density			
City	534/830 (64.3)	reference	reference
Province	975/1385 (70.4)	1.19 (0.98, 1.44)	1.20 (0.99, 1.46)

*aOR (95% CI)* adjusted odds ratios (95% confidence interval)

^*^P-value <0.05, ^**^ *P*-value <0.001

^a^All unknown (missing) variables were included either merely missing or “no response” on purpose.

Multiple logistic regression model was performed in Model 1 and Model 2

^b^Model 1, adjusted for age (65–69, 70–74, 75–79, and ≥80 years), family structure (with someone or others, alone, unknown), educational attainment (≤9, 10–12, ≥13, and unknown years), economic difficulties (yes, no, and unknown), pension (National Pension, Employees’ Pension, others or nothing, and unknown), automobile access (yes / no), interest in health topics (yes, no, and unknown), self-rated health (good, poor, and unknown), smoking (never, past, current, and unknown), social participation (yes / no), and population density (city / province)

^c^Model 2, Model 1 + total score of Kihon checklist (<7, ≥7, and unknown)

^d^These results were indicated 2, 093 men among those who were ‘living with someone or others’ in the family structure.

**Table 4:** The adjusted odds ratios for physical check-up non-participation per sociodemographic status variables among older women

	Non-participation	Model 1 ^b^	Model 2 ^c^
	/ total n (%)	aOR (95% CI)	aOR (95% CI)
Age (years)			
65–69	543/789 (68.8)	reference	reference
70–74	439/682 (64.4)	0.77 (0.61, 0.97)^*^	0.76 (0.60, 0.95)^**^
75–79	387/560 (69.1)	0.88 (0.68, 1.13)	0.84 (0.65, 1.09)
≥80	490/585 (83.8)	1.99 (1.49, 2.66)^*^	1.86 (1.38, 2.52)^**^
Social status			
Family structure			
With someone or others	1454/2048 (71.1)	reference	reference
Alone	243/361 (67.3)	0.78 (0.60, 1.01)	0.78 (0.60, 1.01)
Unknown ^a^	162/207 (78.3)	1.28 (0.88, 1.85)	1.26 (0.87, 1.82)
Living with spouse ^d^			
Yes	572/806 (71.0)	reference	reference
No	770/1112 (69.2)	1.09 (0.88, 1.35)	1.10 (0.89, 1.36)
Unknown	274/337 (81.3)	2.30 (1.33, 3.97)^*^	2.31 (1.33, 3.97)^*^
Alone at home ^d^			
Rarely	276/362 (76.2)	reference	reference
Frequently	1078/1555 (69.3)	0.67 (0.51, 0.88)^*^	0.67 (0.50, 0.88)^*^
Unknown	262/338 (77.5)	0.92 (0.56, 1.51)	0.92 (0.56, 1.52)
Educational attainment (years)			
≥13	270/401 (67.3)	reference	reference
10–12	793/1175 (67.5)	0.95 (0.73, 1.23)	0.94 (0.73, 1.21)
≤9	531/692 (76.7)	1.26 (0.94, 1.69)	1.24 (0.93, 1.67)
Unknown	265/348 (76.2)	1.05 (0.73, 1.49)	1.00 (0.70, 1.44)
Economic status			
Economic difficulty			
No	585/857 (68.3)	reference	reference
Yes	1156/1598 (72.3)	1.18 (0.97, 1.44)	1.18 (0.97, 1.43)
Unknown	118/161 (73.3)	1.13 (0.73, 1.74)	1.10 (0.71 1.70)
Pension			
Mutual or welfare	537/777 (69.1)	reference	reference
National	1163/1638 (71.0)	1.05 (0.87, 1.29)	1.05 (0.86, 1.28)
Nothing or others	93/110 (84.6)	1.49 (0.84, 2.65)	1.47 (0.83, 2.61)
Unknown	66/91 (72.5)	0.98 (0.56, 1.72)	0.98 (0.56, 1.72)
Current employment			
Working	191/291 (65.6)	reference	reference
Not working	1485/2080 (71.5)	1.10 (0.83, 1.46)	1.10 (0.83, 1.46)
Unknown	183/245 (74.7)	1.18 (0.77, 1.80)	1.15 (0.76, 1.76)
Automobile access			
Yes	1211/1730 (70.0)	reference	reference
No	648/886 (73.1)	1.09 (0.89, 1.33)	1.08 (0.88, 1.32)
Health conscious			
Interest in health topics			
Yes	1672/2394 (69.8)	reference	reference
No	140/161 (87.0)	1.81 (1.10, 2.97)^*^	1.78 (1.09, 2.93)^*^
Unknown	47/61 (77.1)	1.10 (0.58, 2.10)	1.04 (0.54, 1.99)
Self-rated health			
Good	1396/2029 (68.8)	reference	reference
Bad	3987/484 (80.0)	1.19 (0.92, 1.55)	1.14 (0.87, 1.50)
Unknown	76/103 (73.8)	0.61 (0.35, 1.04)	0.58 (0.34, 1.01)
Smoking status			
Never	1547/2213 (69,9)	reference	reference
Past	111/156 (71.2)	0.98 (0.67, 1.44)	0.99 (0.68, 1.45)
Current	87/101 (86.1)	2.71 (1.50, 4.91)^*^	2.71 (1.50, 4.90)^*^
Unknown	114/146 (78.1)	1.13 (0.70, 1.81)	1.08 (0.67, 1.74)
Community activities			
Yes	1062/1694 (62.7)	reference	reference
No	8797/922 (86.4)	3.34 (2.66, 4.20)^**^	3.30 (2.62, 4.15)^**^
Population density			
City	661/958 (69.0)	reference	reference
Province	1198/1658 (72.3)	1.08 (0.90, 1.31)	1.07 (0.89, 1.29)

*aOR (95% CI)* adjusted odds ratios (95% confidence interval)

^*^P-value <0.05, ^**^ *P*-value <0.001

^a^All unknown (missing) variables were included either merely missing or “no response” on purpose.

Multiple logistic regression model was performed in Model 1 and Model 2

^b^Model 1, adjusted for age (65–69, 70–74, 75–79, and ≥80 years), family structure (with someone or others, alone, unknown), educational attainment (≤9, 10–12, ≥13, and unknown years), economic difficulties (yes, no, and unknown), pension (National Pension, Employees’ Pension, others or nothing, and unknown), automobile access (yes / no), interest in health topics (yes, no, and unknown), self-rated health (good, poor, and unknown), smoke (never, past, current, and unknown), social participation (yes / no), and population density (city / province)

^c^Model 2, Model 1 + total score of Kihon check list (<7, ≥7, and unknown)

^d^These results were indicated 2,255 women among those who were ‘living with someone or others’ in the family structure.

Whereas in women, higher age-groups (≥80 years) had a significantly higher aOR of non-participation compared to that of the lowest age group in Model 2 (Table 4). The ‘alone’ in family structure showed an inverse association of non-participation than ‘with someone or others’, but not significant (aOR = 0.78, 95% CI: 0.60, 1.01) (Table 4). The aOR of non-participation for individuals who responded with ‘frequently’ to living alone at home was significantly lower than those who responded with ‘rarely’ (aOR = 0.67, 95% CI: 0.50, 0.88). Having no interest in health topics and current smoking status in health conscious both exhibited a positive relationship to non-participation, compared to health topics interest and having never smoked (aOR = 1.78, 95% CI: 1.09, 2.93 in ‘No’ of interest in health topics; aOR = 2.71, 95% CI: 1.50, 4.90 in ‘current’ of smoking status). The non-engagement in community activities, ‘No’ group, showed a positive relationship with non-participation, compared to the engagement group (aOR = 3.30, 95% CI: 2.62, 4.15).

Following results suggesting an association between frailty assessed by KCL and non-participation among men (see Table 5) and women (see Table 6), lower level of IADL and physical function/strength were positively associated with non-participation than higher levels in both men and women (men [IADL] aOR = 1.35, 95% CI: 1.01, 1.82; [physical function/strength] aOR = 1.40, 95% CI: 1.03, 1.91; women [IADL] aOR = 2.42, 95% CI: 1.60, 3.64; [physical function/strength] aOR = 1.36, 95% CI: 1.07, 1.73) (Tables 5 and 6). In Model 2, these associations remained significant even after adjusting for all KCL items, with the exception of IADL among men. Furthermore, the higher level of socialization in men showed a significant, inverse association with non-participation (aOR = 0.76, 95% CI: 0.60, 0.96).

**Table 5:** The adjusted odds ratios of physical checkup non-participation per Kihon Checklist (KCL) scores in older men.

	Non-participation	Model 1 ^b^	Model 2 ^c^
	/ total n (%)	aOR (95% CI)	aOR (95% CI)
Total KCL			
<7	671/1061 (63.2)	reference	
≥7	343/491 (69.9)	0.87 (0.66, 1.13)	
Unknown	495/663 (74.7)	1.26 (0.96, 1.64)	
IADL			
<3	1102/1705 (64.6)	reference	reference
≥3	311/390 (79.7)	1.35 (1.01, 1.82)^*^	1.33 (0.98, 1.81)
Unknown	96/120 (80.0)	1.60 (0.84, 3.04)	1.37 (0.70, 2.66)
Physical function/strength			
<3	1075/1651 (65.1)	reference	reference
≥3	262/336 (78.0)	1.40 (1.03, 1.91)^*^	1.41 (1.02, 1.95)^*^
Unknown	172/228 (75.4)	1.22 (0.86, 1.74)	1.13 (0.75, 1.72)
Malnutrition			
<2	1225/1844 (66.4)	reference	reference
2	36/47 (76.6)	1.13 (0.55, 2.33)	1.18 (0.56, 2.46)
Unknown	248/324 (76.5)	1.24 (0.91, 1.69)	1.21 (0.87, 1.68)
Oral function			
<2	1048/1578 (66.4)	reference	reference
≥2	377/529 (71.3)	0.98 (0.77, 1.24)	0.91 (0.71, 1.17)
Unknown	84/108 (77.8)	1.08 (0.65, 1.81)	0.80 (0.45, 1.44)
Socialization			
<1	960/1439 (66.7)	reference	reference
≥1	487/695 (70.1)	0.85 (0.68, 1.06)	0.76 (0.60, 0.96)^*^
Unknown	62/81 (76.5)	1.02 (0.57, 1.82)	0.75 (0.39, 1.44)
Memory			
<1	780/1214 (64.3)	reference	reference
≥1	642/893 (71.9)	1.13 (0.93, 1.39)	1.10 (0.90, 1.36)
Unknown	87/110 (80.6)	1.47 (0.86, 2.51)	1.27 (0.69, 2.34)
Mood			
<2	911/1413 (64.5)	reference	reference
≥2	399/547 (72.9)	1.11 (0.87, 1.42)	1.12 (0.86, 1.45)
Unknown	199/255 (78.0)	1.96 (1.28, 3.01)^*^	1.93 (1.24, 3.00)^*^

*IADL* instrumental activities of daily living; aOR (95% CI), adjusted odds ratios (95% confidence interval)

^*^P-value <0.05, ^**^ *P*-value <0.001

^a^All unknown (missing) variables were included either merely missing or “no response” on purpose.

Multiple logistic regression model was performed in Model 1 and Model 2

^b^Model 1, age (65–69, 70–74, 75–79, and ≥80 years), family structure (with someone or others, alone, and unknown), educational attainment (≤9, 10–12, ≥13, and unknown years), economic difficulties (yes, no, and unknown), pension (National Pension, Employees’ Pension, others or nothing, and unknown), automobile access (yes / no), interest in health topics (yes, no, and unknown), self-rated health (good, poor, and unknown), smoke (never, past, current, and unknown), social participation (yes / no), and population density (city / province)

^c^Model 2, Model 1 + 7 items of Kihon Checklist (< / ≥ with each cutoff point, and unknown)

**Table 6:** The adjusted odds ratios of physical checkup non-participation per Kihon Checklist (KCL) scores in older women

	Non-participation	Model 1 ^b^	Model 2 ^c^
	/ total n (%)	aOR (95% CI)	aOR (95% CI)
Total KCL			
<7	725/1138 (63.7)	reference	
≥7	467/595 (78.5)	1.14 (0.87, 1.49)	
Unknown	667/883 (75.5)	1.21 (0.95, 1.53)	
IADL			
<3	1404/2098 (67.0)	reference	reference
≥3	310/341 (90.9)	2.42 (1.60, 3.64)^**^	2.28 (1.50, 3.46)^**^
Unknown	145/177 (81.9)	1.79 (1.11, 2.89)^*^	1.63 (0.99, 2.67)
Physical function/strength			
<3	1075/1614 (66.6)	reference	reference
≥3	555/694 (80.0)	1.36 (1.07, 1.73)^*^	1.28 (1.00, 1.65)^*^
Unknown	229/308 (74.4)	1.11 (0.81, 1.52)	0.92 (0.65, 1.30)
Malnutrition			
<2	1466/2114 (69.4)	reference	reference
2	38/52 (73.1)	0.76 (0.39, 1.46)	0.78 (0.40, 1.53)
Unknown	355/450 (78.9)	1.32 (1.00, 1.74)^*^	1.25 (0.93, 1.67)
Oral function			
<2	1311/1887 (69.5)	reference	reference
≥2	433/587 (73.8)	0.85 (0.68, 1.08)	0.80 (0.63, 1.03)
Unknown	115/142 (81.0)	1.33 (0.83, 2.13)	1.16 (0.69, 1.94)
Socialization			
<1	995/1507 (66.0)	reference	reference
≥1	788/1013 (77.8)	1.23 (1.00, 1.51)	1.17 (0.94, 1.46)
Unknown	76/96 (79.2)	1.47 (0.84, 2.58)	1.42 (0.78, 2.59)
Memory			
<1	1095/1602 (68.4)	reference	reference
≥1	668/888 (75.2)	0.98 (0.80, 1.20)	0.92 (0.75, 1.14)
Unknown	96/126 (76.2)	1.06 (0.66, 1.70)	0.85 (0.51, 1.42)
Mood			
<2	1008/1508 (66.8)	reference	reference
≥2	557/736 (75.7)	1.06 (0.84, 1.33)	0.99 (0.78, 1.26)
Unknown	294/372 (79.0)	1.29 (0.92, 1.81)	1.15 (0.81, 1.64)

*IADL* instrumental activities of daily living; aOR (95% CI), adjusted odds ratios (95% confidence interval)

^*^P-value <0.05, ^**^ *P*-value <0.001

^a^All unknown (missing) variables were included either merely missing or “no response” on purpose.

Multiple logistic regression model was performed in Model 1 and Model 2

^b^Model 1, age (65–69, 70–74, 75–79, and ≥80 years), family structure (with someone or others, alone, and unknown), educational attainment (≤9, 10–12, ≥13, and unknown years), economic difficulties (yes, no, and unknown), pension (National Pension, Employees’ Pension, others or nothing, and unknown), automobile access (yes / no), interest in health topics (yes, no, and unknown), self-rated health (good, poor, and unknown), smoke (never, past, current, and unknown), social participation (yes / no), and population density (city / province)

^c^Model 2, Model 1 + 7 items of Kihon Checklist (< / ≥ with each cutoff point and unknown)

The high significant odds ratios for non-participation were observed on unknown variables concerning living with a spouse among both genders educational attainment for men; KCL mood subscore in Model 1 for men; and KCL malnutrition subscore in Model 1 for women, and a negative association with unknown variables was observed in “alone at home” for men. The results excluding unknown variables were not different in the current results with unknown variables but were statistically unclear in some part of the results (data not shown).

## Discussion

To our knowledge, only a few studies have examined what characteristics distinguish non-participants in face-to-face health and physical checkup due to the of lack of detailed data on non-participants. We found that older Japanese adults who were non-participants in a community conducted physical checkup had poorer sociodemographic backgrounds, in addition to greater frailty as indicated by the IADL and physical functioning/strength and aging in women. Specifically, an increase in each of the following factors was linked to a 1.32–3.30-fold increase of non-participation: (for men) lower educational attainment, being on the National Pension (versus the Employees’ Pension), lack of automobile access, poor self-rated health; (for both sexes) no interest in health topics, current smoking, and lack of participation in community activities. On the other hand, for both sexes spending alone at home frequently while living with someone or other family structure was associated with a 0.53–0.67-fold decrease in non-participation was compared to those who were rarely alone at home. Furthermore, when IADL and physical functioning/strength were at a low/impaired level, the odds ratio of non-participation indicated a 1.35–2.42-fold increase compared to those who were at a higher/less impaired level, for both sexes.

To assess consistency, our current findings were compared with other previous research. The overall participation rates were higher in the current study (31.9% in men, 28.9% in women) as compared to rates in five similar studies (range 1.4–16.2%) reported from the previous review [11]. In our study, all individuals eligible to participate in the checkup had already responded to a mail survey at baseline, which may have driven our relatively higher participation rates [11].

Interestingly, we found that when individuals living with someone or other family structure were nevertheless often alone at home, these individuals were in fact relatively more likely to attend the physical checkup. One reason cohabiting individuals who nevertheless spend much time alone might attend the checkup would be for social exchange and conversation with neighbors or staff. It should also be noted that the opportunity to participate in physical checkups may help individuals who were low in socialization to go outside. The physical checkup is expected to be a significant opportunity for social exchange and preventing the situation of being housebound among older residents.

The current results were in the line with the previous research observing an association between poor socioeconomic status and non-participation in health programs and health checkups in middle age and older populations [14, 25–27]. For instance, lower educational attainment has been consistently found to predict non-participation in sports groups among Japanese older people [14]. While, a previous study was no association between educational level and participation in a health checkup for Germans aged 35 years or older [27]. The relatively large number of women with low educational attainments in our study may have attenuated the impact of this association in the current study. Qualitative research has found that possible barriers to the participation in physical activity programs include unavailability of access, cost, convenience of physical activity programs, and physical limitations due to health conditions [25]. The current results suggest that non-participants were more likely to reside in provincial area and men who were unlikely to use automobiles and thus could expect poor accessibility, thereby depriving them of the opportunity to participate in the physical checkup. These findings indicate that economic status impacts participation in health programs more for older Japanese men than for women.

Health consciousness, including self-rated health, interest in health topics, and smoking status may distract older people from acting out healthy behaviors (i.e., participation in physical activity program). A similar trend was observed in prior research, in which a significant association emerged between poor self-rated health and lower attendance of a health checkup among Australians aged 20 years or older (population aged 65 years or older was 44.5% in men) [26]. An association between social participation and a high level of self-rated health has also been found previously [28]. Participation in a community based health program can be regarded as a type of social participation [28–30]. Thus, it could be that individuals with poor health consciousness have low motivation for social participation, and thus not participated in a physical checkup.

In a cross-sectional study, it was found that participation in community and social activities was significantly associated with engaging in physical fitness study [31]. Participants who engaged in a social activity had higher levels of locomotive function [31]. A longitudinal study also found that social participation among older Japanese people, including social activity, was associated with a lower risk of functional disability [29, 30]. It may be that participants who do not participate in social activities hesitated to participate in the physical checkup due to their low level of physical functioning.

It has been reported that characteristics of participants differed from the strengths and the contents of physical activity programs (i.e., gardening or yard work, walking, and sports or exercise) according to gender and functional health [32]. Qualitative research also suggests that physical limitations due to health conditions were a potential barrier to participation in physical activity programs [25]. Literature reported the negative association between regular participation in sports groups and IADL level [14]. Indeed, our study found a significant association between participating in no community activities and being a non-participant. Although this study cannot determine causality, it links anxiety related to low physical function level to both lower participation in community activities and non-participation in physical checkups. In addition, aging, which was associated with frailty, may more predict non-participation in a physical checkup in women as compared to that in older men, according to the present results. It is said that older women get a muscle damage and low grip strength with aging more easily than older men [33]. These weaknesses of muscle with aging would hinder older women in participation of physical checkup. In the current study, it might have been hard for more frail individuals (as indicated by IADL and physical functioning/strength) and frail women due to aging to engage in several tests of physical fitness over one hour. In addition, accessing the location of the program may have been difficult for frail individuals.

However, our findings are also inconsistent with some of those reported in previous studies. While we did not find a significant relationship between unemployment and participation, Yamakita et al. reported a significant association of unemployment with non-participation in sports activities [14]. However, while not statistically significant, among men in the study, the effect was in the same direction (i.e., unemployment predicting non-participation). Among women, limited number of participants who were ‘working’ in the current employment might result in the unclarified association. In our study, current mood was assessed by the KCL with 5 items, while Yamakita et al. assessed depression with the 15-item Geriatric Depression Scale–15 [14]. Therefore, differences in measurement may explain the different associations.

It should be noted that part of unknown variables were positively associated with the non-participation in this study. Although we could not clarify the reason, the missing variables may have a link with the latent background of non-participation.

The possible assessment of our findings could be described as below. Cornwell et al. indicate that social isolation consists of a lack of social support and feelings of loneliness [34, 35]. When older people feel loneliness due to poor sociodemographic status and physical frailty, fears of social participation may hinder their motivation to participate in a physical activity program. To prevent health inequality, it is necessary to enhance social support of non-participants so that they can have the opportunity to attend health programs.

The main strength of this study is that our findings indicated that poor sociodemographic status and physical frailty may cause non-participation, as the physical checkup was implemented with over a six-month time lag from the baseline survey.

Our study had several limitations. First, all eligible subjects were responders at the baseline survey (73.2% of total residents). This study cannot clarify associations between personal characteristics and non-participation for the types of individuals who did not respond to the baseline survey (26.8% of total residents). Second, our sample was ascertained from a specific Japanese community, and our findings may not generalize to other older population. Third, we used a single arm for recruiting, and did not compare the different types of recruiting methods, which may be a confounding factor. Finally, there is a possibility that participants of physical checkup were authorized people (e.g, working staff, city officers, and their family or their relatives), which may be a confounding factor that describes the characteristics of participants. However, there were few authorized people and their impact on the results may be low. Because the impact of the number of research staffs on participation rates may low, we did not mention in the present study. The interesting finding that the participation rate in men was higher than in women may indicate a need to explore differences in recruiting methods and specifics of particular programs.

## Conclusions

In conclusion, a poor sociodemographic background and physical frailty may predict non-participation in community based physical activity programs in community-dwelling older people. To develop a health program considering the role of social exchange may contribute to improve the participation rates. At the same time, we should build health program with considering the level of aging and physical frailty to increase the participation. To prevent health inequality, non-participants should have opportunity to receive social support to participate a community-based health program without barriers.

## Additional files

Additional file 1 **Table S1.** Needs in the Sphere of Daily Life questionnaire. (DOCX 19 kb)

Additional file 2 **Table S2.** The Kihon-Checklist with 25 questions. (DOCX 20 kb)

## Abbreviations

CI: Confidence interval
aOR: Adjusted odds ratio
IADL: Instrumental activities of daily living
KCL: Kihon Checklist
NSDL: Needs in the Sphere of Daily Life
SD: Standard deviation
