# Supplementary material for: Sociodemographic and physical predictors of non-participation in community based physical checkup among older neighbors: a case-control study from the Kyoto-Kameoka longitudinal study, Japan
Source: BMC Public Health. 2018 May 2;18:568. doi: 10.1186/s12889-018-5426-5 (PMC5930753; doi:10.1186/s12889-018-5426-5)
Supplement: Supplementary file 1 — Table S1. Needs in the Sphere of Daily Life questionnaire. (DOCX 19 kb) [file 12889_2018_5426_MOESM1_ESM.docx]

**Additional file 1: Table S1.** Needs in the Sphere of Daily Life questionnaire

| **Questionnaire for NSDL survey** |
| --- |
| **Social status** |
| 1. With whom do you live? |
| 1. If you live with someone, do you live with your spouse? |
| 1. How frequently do you stay at home alone all day if you live with someone? |
| 1. How many years did you go to school when you were between six and 30 years old? |
| **Economic status** |
| 1. How is your economic situation? |
| 1. Please select the type of pension you receive. |
| 1. Do you currently work to get a salary? |
| 1. Do you use automobiles, regardless of driving? |
| **Health consciousness** |
| 1. Are you interested in books or magazines about health? |
| 1. Do you feel that you are usually healthy? |
| 1. Do you smoke? |
| **Community activities** |
| 1. Please select all community activities you attend. |
