# Supplementary material for: Sociodemographic and physical predictors of non-participation in community based physical checkup among older neighbors: a case-control study from the Kyoto-Kameoka longitudinal study, Japan
Source: BMC Public Health. 2018 May 2;18:568. doi: 10.1186/s12889-018-5426-5 (PMC5930753; doi:10.1186/s12889-018-5426-5)
Supplement: Supplementary file 2 — Table S2. The Kihon-Checklist with 25 questions. (DOCX 20 kb) [file 12889_2018_5426_MOESM2_ESM.docx]

**Additional file 2: Table S2.** The Kihon-Checklist with 25 questions

| **Kihon-Checklist (KCL)** |
| --- |
| **Instrumental activities of daily living** |
| 1) Do you take the bus or train by yourself? |
| 2) Do you go shopping to buy daily necessities by yourself? |
| 3) Do you manage your own deposits and savings at the bank? |
| 4) Do you sometimes visit your friends? |
| 5) Do you turn to your family or friends for advice? |
| **Physical function/strength** |
| 6) Do you normally climb stairs without using handrails or wall for support? |
| 7) Do you normally stand up from chairs without any aid? |
| 8) Do you normally walk continuously for 15 minutes? |
| 9) Have you experienced a fall in the past year? |
| 10) Do you have a fear of falling while walking? |
| **Malnutrition** |
| 11) Have you lost 2 kg or more in the past 6 months? |
| 12) Is your body mass index (kg/m^2^) less than 18.5 |
| **Oral function** |
| 13) Do you have any difficulty eating tough foods compared to 6 months ago? |
| 14) Have you choked on your tea or soup recently? |
| 15) Do you often experience having a dry mouth? |
| **Socialization** |
| 16) Do you go out at least once a week? |
| 17) Do you go out less frequently compared to last year? |
| **Memory** |
| 18) Do your family or your friends point out your memory loss? e.g. “You always ask the same question over and over again.” |
| 19) Do you make a call by looking up phone number? |
| 20) Do you find yourself not knowing today’s date? |
| **Mood** |
| 21) In the last two weeks, have you felt lack of fulfillment in your life? |
| 22) In the last two weeks, have you felt a lack of joy when doing the things you used to enjoy? |
| 23) In the last two weeks, have you felt difficulty in doing what you could do easily before? |
| 24) In the last two weeks, have you felt helpless? |
| 25) In the last two weeks, have you felt tired without a reason? |
